# Non-Conduit Repair of Truncus Arteriosus

**DOI:** 10.21470/1678-9741-2022-0029

**Published:** 2023

**Authors:** Yiğit Kılıç, Onur Doyurgan, Ahmet Kuddusi İrdem, Özlem Gül, Dilek Borakay, Bedri Aldudak

**Affiliations:** 1 Department of Pediatric Cardiac Surgery, Dr. Gazi Yasargil Training and Research Hospital, Diyarbakir, Turkey.; 2 Department of Pediatric Cardiology, Dr. Gazi Yasargil Training and Research Hospital, Diyarbakir, Turkey.

**Keywords:** Truncus Arteriosus, Heart Ventricles, Pulmonary Artery, Infant, Intensive Care Units

## Abstract

**Introduction:**

The procedure of choice for treatment of truncus arteriosus is one-stage repair within the first few months of life. Establishing right ventricle-pulmonary artery direct continuity without conduit can be a good alternative in the absence of valved conduits in developing centers.

**Methods:**

Between January 2021 and June 2021, a total of five patients (three males, two females) underwent definitive repair of truncus arteriosus without an extracardiac conduit. We used the Barbero-Marcial technique to allow age-related growth, eliminate the risk of conduit-related complications, and to avoid forcing a conduit to place in a very small mediastinal space.

**Results:**

The patients’ mean age was 31.2 days (11-54 days). Their mean bodyweight was 3.2 kg (2.7-3.8kg). Mean postoperative intensive care unit stay was 39.6 days (7-99 days). There were two mortalities in the intensive care unit on postoperative days 12 and 61 due to lung-related problems. The remaining three cases’ mean ventilation time was 15.6 days (8-22 days).

**Conclusion:**

Having access to a valved conduit is still challenging for some centers, and the non-conduit repair technique defined by Barbero-Marcial can be a successful, life-saving alternative easy for young surgeons to perform in newly based centers.

## INTRODUCTİON

Truncus arteriosus (TA) is a relatively rare congenital heart anomaly which constitutes 1-3% of all congenital heart diseases^[^12^]^. A variety of well-known techniques has been used to establish the right ventricle (RV)-pulmonary artery (PA) continuity^[^3^, ^4^, ^5^]^. Surgical repair of TA is still challenging, but, basically, it has been performed with a conduit or direct anastomosis as described by Barbero-Marcia et al.^[^6^]^ so far. Bovine jugular venous valved conduit (Contegra®) came in use in 1999 andit is widely used all around the world as it has a soft tissue, good hemostatic results in neonates, and little pressure on surrounding vital structures^[^7^, ^8^, ^9^]^. Regardless of which conduit is used, there is always the risk of stenosis, valve failure, and these conduits do not have growth potential. These complications result in reoperation. Every reoperation to change these conduits increases the risk of mortality and morbidity. Also, there are traditional factors restricting conduit usage or problems in having access to these materials in some countries. In recent years, early mortality was described as low as 5-10% in some centers^[^10^]^. Another TA study reported in-hospital death as 9-11%^[^11^, ^12^]^. But it is still high. As we had no valved conduits of suitable size for newborns and small infants in our hospital, by establishing RV-PA direct continuity without conduit we aimed to allow age-related growth, eliminate the risk of conduit-related complications, and to avoid forcing a conduit to place in a very small mediastinal space, especially in a small weight baby. In this manner, we used the technique described by Barbero-Marcial et al.^[^6^]^.

## METHODS

Between January 2021 and June 2021, a total of five patients (three males, two females; median age of 31.2 days, ranging from 11 to 54 days) underwent definitive repair of TA without an extracardiac conduit. Their average weight was 3.2 kg, ranging from 2.7 to 3.8 kg; and the mean body surface area was 0,21 kg/m2. According to the Collett-Edwards classification, all patients were type 1 TA. Echocardiographies performed preoperatively showed that all the patients had a large subtruncal ventricular septal defect (VSD), and none of the cases had severe truncal valve insufficiency. The study protocol was approved by Diyarbakir Gazi Yasargil Training and Research Hospital Ethics Committee (Date: 14.01.2022, Number: 2). A written informed consent was obtained from each parent. The study was conducted in accordance with the principles of the Declaration of Helsinki.

### Technique

**Fig. 1:**
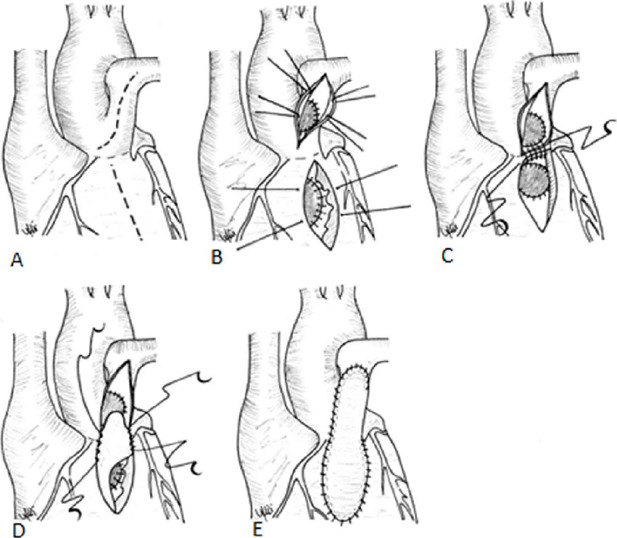
A) A longitudinal incision was made into the anterosuperior aspect of the left pulmonary artery. Then, the incision was extended vertically towards the left sinus of Valsalva, leaving a few millimeters of tissue over the truncal root for suturing. Superiorly, the incision was extended towards the left pulmonary orifice. B) Being careful with the left coronary ostium, right pulmonary artery orifice, and truncal valve cusps, new aortic and pulmonary roots were separated from each other using a glutaraldehyde-treated autologous pericardium as an aortopulmonary window patch. Right ventriculotomy incision was made parallel to the first incision, and closure of VSD was done with running suture technique using a Dacron patch through this ventriculotomy. C) The posterior wall was created by suturing left pulmonary artery directly to the left superior oblique margin of the right ventriculotomy with running suture technique. Anastomosis is reinforced with pericardial pledgeted 7-0 polypropylene sutures (not shown). D) A 0,1-mm polytetrafuoroethylene patch was prepared as a monocusp and sutured directly to the new right ventricular outflow tract. E) The anterior wall was constructed by suturing an autologous pericardial patch.

After median sternotomy, aorta and PAs were dissected. Cardiopulmonary bypass was initiated following standard aortobicaval cannulation. Right and left PAs were fully mobilized and snared. Under moderate hypothermia, after cross-clamping the aorta, the heart was arrested using del Nido cardioplegia. A longitudinal incision was made into the anterosuperior aspect of the left PA. Then, the incision was extended vertically towards the left sinus of Valsalva, leaving a few millimeters of tissue over the truncal root for suturing. Superiorly, the incision was extended towards the left pulmonary orifice. Being careful with the left coronary ostium, right PA orifice, and truncal valve cusps, new aortic and pulmonary roots were separated from each other using a glutaraldehyde-treated autologous pericardium as an aortopulmonary window patch. Right ventriculotomy incision was made parallel to the first incision, and closure of VSD was done with running suture technique using a Dacron patch through this ventriculotomy. The posterior wall was created by suturing left pulmonary artery directly to the left superior oblique margin of the right ventriculotomy with running suture technique. Anastomosis is reinforced with pericardial pledgeted 7-0 polypropylene sutures. A 0.1-mm polytetrafuoroethylene patch was prepared as a monocusp and sutured directly to the new right ventricular outflow tract. The anterior wall was constructed by suturing an autologous pericardial patch. Atrial septum was fenestrated 2 to 3 mm in size to prevent right ventricular dysfunction. The rest of the operation was performed as usual (Figure 1).

## RESULTS

Conduit-free surgery for TA was performed in five patients (three males, two females) between January 2021 and June 2021. Our policy is to repair TA within two months after birth, preferably in the neonatal period, when the baby weighs over 2.5 kg, after their second/third week of life. The patients’ mean age was 31.2 days (11-54 days). Their mean bodyweight was 3.2 kg (2.7-3.8 kg). The Collett-Edwards classification of TA was type I in all patients. None of them had coronary anomaly or valve regurgitation more than mild. Truncal valve plasty wasn’t performed in any of the patients. The sternum was left open in one patient, and chest closure was successfully performed in this case on postoperative day five. The mean postoperative intensive care unit (ICU) stay was 39.6 days (7-99 days). There were two mortalities in the ICU on postoperative days12 and 61 due to a prolonged course of mechanica ventilation and lung-related problems. The remaining three cases mean ventilation time was 15.6 days (8-22 days). One patient was treated with sildenafil because of mild pulmonary hypertension.

## DİSCUSSİON

The procedure of choice for treatment of TA is one-stage repair within the first few months of life. Reinterventions are inevitable for valved conduit patients as the patient grows up or the conduit degenerates. But the non-conduit repair technique of Barbero-Marcial gives the patient chance of natural growth. Also, one can easily use this technique in patients with low birth weight who have really limited mediastinal space and close the sternum. We only had one delayed sternal closure. The weight of our lighter patient was 2.7 kg at the time of the procedure.

This technique has its own limitations. The pulmonary trunk is pulled down to the right ventricular incision for direct anastomosis and this may cause the posterior wall of the new pulmonary trunk to be short and narrow. Barbero-Marcial et al.^[^13^]^ modifed the technique by interposing the left atrial appendage between RV and pulmonary trunk to overcome this situation. Another imitation is the new location of the pulmonary bifurcation. It is closer to the right ventricular incision more than usual and this may result in difficulties in future prosthetic valve insertion^[^14^]^. In our newly based center, while dealing with complex cases, we also have difficulties in having access to small-size valved conduits for TA repair. Under these circumstances, non-conduit repair appears to be the best choice. All of our cases were type 1 TA with normal coronary anatomy. As none of them had more than grade I truncal valve insufficiency or 30 mmHg gradient, there was no need for truncal valve repair in any of the cases.We had no operative death. There were two postoperative deaths on days 12 and 61 because of respiratory system-related problems. These patients with type I TA were our initial cases, and low experience in our ICU dealing with small weight babies with complex anomalies was the main challenging factor. Two of the patients also had genetic anomalies. One of the patients had a tracheostomy procedure and was discharged successfully.

Our follow-up echocardiographies showed that the patients had good postoperative myocardial function and they had mild or no pulmonary insufficiency.

Since one of these three cases was discharged abroad, it could not be followed up regularly. Cardiac functions of the remaining two cases were good at the ninth-month control echocardiographies. While significant pulmonary stenosis or insufficiency was not detected in one case, mild pulmonary insufficiency and moderate pulmonary stenosis were detected in the other.

### Limitations

As we don’t have 5-10 years of long-term follow-up, we haven’t faced the limitations mentioned in the literature during long-term follow-up.

## CONCLUSİON

Surgical repair of TA is still challenging. Despite improvements in perioperative care, mortality rates are high (9-11%)^[^11^,^12^]^. Having access to a valved conduit is still challenging for some centers, and the non-conduit repair technique defined by Barbero-Marcial can be a successful, life-saving alternative easy for young surgeons to perform in newly based centers.
